# Mosquitoes, Infectious Diseases, and Cancer: A Connection to Study?

**DOI:** 10.3390/ijerph16234859

**Published:** 2019-12-03

**Authors:** Carlos Brisola Marcondes, Giovanni Benelli

**Affiliations:** 1 Departamento de Microbiologia, Imunologia e Parasitologia, Centro de Ciências Biológicas, Universidade Federal de Santa Catarina, Florianópolis 88040-900, SC, Brasil; 2 Department of Agriculture, Food and Environment, University of Pisa, via del Borghetto 80, 56124 Pisa, Italy

**Keywords:** *Aedes aegypti*, dengue, hypersensitivity, immunosuppression, One Health, *Plasmodium* parasites, vector-borne diseases

## Abstract

Mosquitoes (Diptera: Culicidae) are vectors of pathogens and parasites of great medical and veterinary relevance. The possible association between mosquitoes, infectious diseases, and cancer has been investigated. Despite its potential importance, there is a severe lack of research data on the topic. Herein, current knowledge, tenuous links, and related challenges on the topic were examined, grouping information under four major hypotheses. The first hypothesis is that the infection of mosquito-vectored parasites, with special reference to *Plasmodium* spp., may lead to cancer. The International Agency for Research on Cancer stated that being infected by *Plasmodium falciparum* malaria in holoendemic areas is probably carcinogenic to humans (group 2A), considering that *P. falciparum* infection is able to reactivate the Epstein–Barr virus, leading to endemic Burkitt lymphoma. Also, malaria was recently associated with a cancer incidence increase in the United States. The second hypothesis is that cancer may be spread directly through mosquito bites: *Aedes* mosquitoes transfer viable tumor cells among vertebrate hosts, even if no plausible mechanisms for these cells to develop cancer into the new host are known. As the third hypothesis, mosquito bites may lead to hypersensitivity, resulting in cancer. Hypersensitivity stimulated by mosquito bites links allergy, oncogenesis, and the Epstein–Barr virus, causing Burkitt lymphoma. One may argue that pathogens transmitted by mosquitoes, such as viruses, may be carcinogenic. However, no detailed research evidences are available to substantiate this last hypothesis. However, despite the intriguing hypotheses outlined above, there is a severe lack of data showing cancer development in organisms exposed to mosquitoes transmitting parasites or pathogens. According to One Health criteria, this benchmark is aimed to outline major questions on this public health issue, stressing the need of multidisciplinary research and discussion.

## 1. Introduction

Mosquitoes (Diptera: Culicidae) have been incriminated as vectors of hundreds of pathogens and parasites, including viruses (e.g., dengue, yellow fever, and Zika viruses), parasitic protozoan species (*Plasmodium* spp. parasitizing mammals and birds), and helminths (e.g., filariae), leading to severe infectious diseases, mostly in tropical and subtropical regions worldwide [1,2,3]. Mosquitoes also cause great annoyance, especially when biting in high numbers [4]. Recently, an association between mosquitoes, infectious diseases, and cancer has been studied, and researchers from the United Kingdom, Germany, Italy, and Sweden have published reviews and a short communication on this issue [5,6,7,8]. Despite the importance of this potential association, there is a substantial lack of recent research data on the topic. Some relevant information is summarized and critically discussed in the paragraphs below, to stimulate debate and further research on this issue. Current knowledge and related challenges on the topic have been grouped under four major hypotheses: (*i*) infection of mosquito-vectored parasites, with special reference to *Plasmodium* spp., may lead to cancer; (*ii*) cancer may be spread directly through mosquito bites; (*iii*) mosquito bites may lead to hypersensitivity, resulting in cancer; and (*iv*) pathogens transmitted by mosquitoes, such as viruses, may be carcinogenic.

## 2. Hypothesis 1: Infection of Mosquito-Vectored Parasites May Lead to Cancer

The impact of selected protozoan and helminth parasites on the spread of different cancers has been widely studied [9,10]. Most of the research work has been done on the carcinogenic role of *Schistosoma haematobium* (Bilharz), *Opisthorchis viverrini* (Poirier) Stiles & Hassall, and *Clonorchis sinensis* (Cobbold) [9], but important knowledge is also available about the association between malaria and carcinogenesis [11], with special reference to endemic Burkitt lymphoma [12,13,14,15]. 

Indeed, a significant geographical association between the latter and holoendemic malaria was firstly discovered by Dr. D. Burkitt in Sub-Saharan Africa [15]. Later, higher endemic Burkitt lymphoma incidence in areas with *P. falciparum* holoendemic transmission was confirmed through multiple correlation studies [13,14]. Further research detailed when and how *Plasmodium* infection can dysregulate the oncogenic Epstein–Barr virus [16,17,18,19,20], which is needed for the pathogenesis of endemic Burkitt lymphoma [21]. 

Based on this, the WHO International Agency for Research on Cancer Monograph Working Group classified the infection with *P. falciparum* parasites in holoendemic areas as “probably carcinogenic to humans” (group 2A) [15,22]. In addition, malaria was associated with an increase in the incidence of cancer in the United States [23]. Avian and simian malaria can also be present in humans, being that their occurrence is underestimated, with a possible association to cancer [5,7].

## 3. Hypothesis 2: Cancer Cells May Be Spread Directly through Mosquito Bites

In a previous study published in *Science*, it was reported that the hamster reticulum cell sarcoma can be transmitted by the bite of *Aedes aegypti* (L.) (Diptera: Culicidae) by the transfer of tumor cells [24] that can remain viable in mosquitoes up to eight hours [24,25]. However, we should point out that there is no plausible mechanism described for these cells to make their way into a new host upon a subsequent blood meal for most known modes of transmission.

## 4. Hypothesis 3: Mosquito Bites May Lead to Hypersensitivity, Resulting in Cancer

The abovementioned association between malaria and cancer in the United States [23] could also be also linked to immunosuppression caused by the infection. High rates of vector biting might lead to immunosuppression, allowing persistent viral infections and cancers to reactivate. In Japan, mosquito bites were found to stimulate hypersensitivity, leading to high fever, malaise, and hepatosplenomegaly. This hypersensitivity, with strong racial predisposition [26], is triggered by a mechanism linked to Epstein–Barr virus infection [26], causing Burkitt lymphoma, allergy, and oncogenesis. Indeed, the host response involves several cells, including lymphocytosis (Natural Killer cells) of CD4+ cells, related to hypersensitivity to bites and oncogenesis with cells containing Epstein–Barr [27]. CD4+ T cells from a hypersensitive patient responded to certain mosquito salivary gland extracts, and these cells could induce reactivation of latent Epstein–Barr virus infection [27]. As stressed above, the development of Burkitt lymphoma can be related to cofactors produced by infection of *Plasmodium* parasites [15]. 

In addition, a significant rise in the number of apoptotic cells, coupled with increased production of tumor necrosis factor alpha (TNFα) in dengue (DEN-2)-infected human monocyte cultures, has been detailed by Espina et al. [28], and the production of TNFα is one of the main factors leading to hemorrhage [29]. Hypersensitivity to mosquito bites (HMB) may also signalize the existence of some neoplasia like mantle cell [30] and marginal zone lymphoma [31].

## 5. Hypothesis 4: Pathogens Transmitted by Mosquitoes May Be Carcinogenic

In the first hypothesis, we focused on the potential role of mosquito-vectored parasites, such as *Plasmodium*, which, according to IARC, may act as carcinogenic agents. Besides parasites, one may argue: what do we really know about the potential carcinogenic role of mosquito-vectored pathogens? Indeed, still on the association between malaria and cancer in the United States [23], it has been hypothesized that *Anopheles* mosquitoes transmit viruses associated with brain cancer [32], or that this association may just reflect common well-established predispositions of certain socioeconomic or geographic groups.

However, screening literature on the possible transmission of carcinogenic pathogens through mosquito biting activity, we faced a severe lack of research data. Notably, mosquitoes can transmit a cocktail of infectious agents, possibly 60 or more, which may come from their breeding places, sometimes highly contaminated by living beings and potentially toxic substances [33]. Besides the possible association of some of these agents to cancer, according to IARC, this cocktail may induce immunosuppression [5]. 

However, two major issues need to be considered. First, none of the known viral etiologies of cancer is vector-borne; most of them are DNA viruses, while most of the vector-borne viruses have an RNA genome [34]. Second, the recent use of next-generation sequencing of nucleic acids from mosquitoes and other vector groups has not reported the presence of oncolytic viruses or their relatives.

## 6. Conclusions

Overall, we critically focused on four hypotheses outlining tenuous links among mosquito bites, parasites and pathogens transmitted by these insects, and cancer. However, despite the intriguing scenarios depicted in the hypotheses and related knowledge highlighted above, experimental data showing cancer development in organisms exposed to mosquitoes transmitting parasites or pathogens are still lacking. 

### Perspectives for Future Research

Research efforts on this issue would be extremely helpful and are urgently needed, especially considering the likely increase of mosquito-borne diseases in the forthcoming years due to the spread of highly invasive vectors linked to global warming and urbanization [35]. We envision that the present article will promote multidisciplinary discussion and research on this issue of high relevance for medical entomology, parasitology, and public health, fitting the One Health criteria, which strongly encourage the cooperation among multiple disciplines, to achieve the best health for humans, animals, and the environment [36,37].
